# Efficacy of SGLT2 Inhibitors Versus Pioglitazone in the Treatment of Non-alcoholic Fatty Liver Disease or Non-alcoholic Steatohepatitis: A Systematic Review

**DOI:** 10.7759/cureus.45789

**Published:** 2023-09-22

**Authors:** Simran Kaur, Vani Sojitra, Anam Zahra, Jhenelle Hutchinson, Oluwafolawemi Adefokun, Parikshit Bittla, Shivana Ramphall

**Affiliations:** 1 Internal Medicine, California Institute of Behavioral Neurosciences & Psychology, Fairfield, USA; 2 Medicine, Bavadia Hospital, Una, IND; 3 Surgery, California Institute of Behavioral Neurosciences & Psychology, Fairfield, USA; 4 Psychiatry, California Institute of Behavioral Neurosciences & Psychology, Fairfield, USA; 5 Medicine, California Institute of Behavioral Neurosciences & Psychology, Fairfield, USA

**Keywords:** nash and sglt2 inhibitor, nonalcoholic fatty liver disease (nafld), nash and steatosis, comparative efficacy, systematic review, pioglitazone, sglt2 inhibitors, non-alcoholic steatohepatitis, non-alcoholic fatty liver disease

## Abstract

Non-alcoholic fatty liver disease (NAFLD) is a complication related to obesity and metabolic syndrome. There are increased incidences of NAFLD/non-alcoholic steatohepatitis (NASH) due to rising obesity and type 2 diabetes mellitus (T2DM). This has resulted in significant morbidity and mortality. The two promising therapeutic agents for treating NAFLD/NASH are sodium-glucose cotransporter 2 (SGLT2) inhibitors and pioglitazone. The reason is their potential to target underlying pathophysiological mechanisms. SGLT2 inhibitors may help treat NAFLD/NASH by reducing insulin resistance and improving glucose control, thereby lowering hepatic fat accumulation and inflammation, although their exact mechanism in this context is still being studied.

This systematic review aims to compare the efficacy of SGLT2 inhibitors and pioglitazone in treating NAFLD/NASH. Major research literature databases were searched, and appropriate keywords were used to find relevant articles published in the last three years. Eighteen studies were critically evaluated using standardized quality assessment tools. Among those, nine studies qualified as medium or high quality and were included in the review. Both SGLT2 inhibitors and pioglitazone showed promising results in improving NAFLD/NASH.

The efficacy outcomes assessed liver fat content, liver enzyme levels, histological improvement, and metabolic parameters. The safety outcomes considered adverse events and cardiovascular events. The conducted review suggests that SGLT2 inhibitors and pioglitazone are potential treatment options for NAFLD/NASH. Having said that, individualized considerations are essential. It includes patient comorbidities, preferences, and overall safety profiles. Further research is needed to assess long-term effects and outcomes. It would provide more definitive evidence of these treatment options’ comparative efficacy and safety for NAFLD/NASH.

## Introduction and background

Non-alcoholic fatty liver disease (NAFLD) is a condition associated with obesity and metabolic syndrome. It is marked by the accumulation of fat in liver cells not related to alcohol consumption [1,2]. The condition in its advanced stage is known as non-alcoholic steatohepatitis (NASH). NAFLD has three stages: hepatic steatosis, NASH without fibrosis, and NASH with fibrosis. Progression is slow, taking years to decades. It is a major health concern that affects approximately 25% of adults globally [3,4]. Lately, due to an increase in obesity and type 2 diabetes mellitus (T2DM), there has been a rise in the occurrence of this disease, which in turn results in high levels of morbidity and mortality [5]. The incidence of NAFLD/NASH is on the rise due to the increase in obesity and T2DM. This is resulting in high levels of morbidity and mortality. Experts predict that NAFLD will become the leading cause of liver disease mortality in the next two decades. Moreover, it is expected to be a major contributor to the demand for liver transplants in the future [5]. Lately, our understanding of the pathogenesis and therapeutic options for NAFLD/NASH has advanced. Therefore, novel interventions are being explored to manage this complex condition effectively.

There have been promising developments in the treatment of NAFLD/NASH with the emergence of sodium-glucose cotransporter 2 (SGLT2) inhibitors and pioglitazone. These therapeutic agents have the potential to target the fundamental pathophysiological mechanisms that are involved in the progression of the disease. SGLT2 inhibitors are primarily used to treat T2DM by inhibiting renal glucose reabsorption, which in turn leads to an increase in urinary glucose excretion and better glycemic control [5]. Besides reducing glucose levels, SGLT2 inhibitors are also known for improving liver fat accumulation and inflammation [6,7]. Pioglitazone, a thiazolidinedione (TZD), is known for increasing insulin sensitivity and reducing systemic insulin resistance. It has effectively improved hepatic steatosis, inflammation, and fibrosis in patients with NAFLD/NASH [8].

As we see the potential benefits of both SGLT2 inhibitors and pioglitazone, we believe that a systematic review is called for to critically evaluate the existing evidence and also compare the efficacy of these two drugs in treating NAFLD/NASH. This systematic review aims to comprehensively assess and summarize the available literature, including randomized controlled trials (RCTs) and observational studies, to determine the comparative effectiveness of SGLT2 inhibitors and pioglitazone in terms of liver histology improvement, liver enzyme normalization, glycemic control, body weight changes, and safety outcomes. This systematic review will contribute to a better understanding of the efficacy and safety of SGLT2 inhibitors and pioglitazone in the treatment of NAFLD/NASH. These findings will help to make informed decisions regarding the efficient pharmacological approach for managing NAFLD/NASH. This could help in the advancement of more targeted therapeutic strategies for this disease.

## Review

Method

We conducted a systematic review and adhered to the guidelines and principles outlined in the Preferred Reporting Items for Systematic Reviews and Meta-Analyses (PRISMA 2020). The results have been reported in accordance with these guidelines [6,9].

Search Sources and Search Strategy

To gather information on the topic, research literature databases and search engines like PubMed, Embase, and Wiley Online Library were utilized. Relevant articles were found by using appropriate keywords.

To find pertinent articles, the databases were searched using the keywords "NAFLD," "SGLT2 inhibitors," and "Pioglitazone." The keywords were combined in different combinations using Boolean operators "AND" and "OR." Table 1 shows the Medical Subject Heading (MeSH) strategy and Table 2 provides the comprehensive search technique using three data sources.

**Table 1:** MeSH strategy NAFLD: Non-alcoholic fatty liver disease; NASH: Non-alcoholic steatohepatitis; SGLT-2: Sodium-glucose cotransporter 2; MeSH: Medical Subject Heading

Mesh strategy
Concept 1 – NAFLD / NASH (“Non-alcoholic Fatty Liver Disease/drug therapy"[Mesh] OR “Non-alcoholic Fatty Liver Disease/therapy"[Mesh])
Concept 2 – SGLT-2 inhibitors (“Sodium-Glucose Transporter 2 Inhibitors/adverse effects"[Mesh] OR “Sodium-Glucose Transporter 2 Inhibitors/pharmacology"[Mesh] OR “Sodium-Glucose Transporter 2 Inhibitors/therapeutic use"[Mesh]))
Concept 3 – pioglitazone (“Pioglitazone/adverse effects"[Mesh] OR “Pioglitazone/pharmacology"[Mesh] OR “Pioglitazone/therapeutic use"[Mesh])
Full mesh strategy (((“Non-alcoholic Fatty Liver Disease/drug therapy"[Mesh] OR “Non-alcoholic Fatty Liver Disease/therapy"[Mesh])) AND (“Sodium-Glucose Transporter 2 Inhibitors/adverse effects"[Mesh] OR "Sodium-Glucose Transporter 2 Inhibitors/pharmacology"[Mesh] OR "Sodium-Glucose Transporter 2 Inhibitors/therapeutic use"[Mesh] )) AND ( "Pioglitazone/adverse effects"[Mesh] OR "Pioglitazone/pharmacology"[Mesh] OR "Pioglitazone/therapeutic use"[Mesh] )

**Table 2:** Database and search strategy using keywords NAFLD: Non-alcoholic fatty liver disease; NASH: Non-alcoholic steatohepatitis; SGLT-2: Sodium-glucose cotransporter 2

S. No	Databases	Keywords	Search Results
1	PubMed	SGLT-2 Inhibitors and NAFLD	116
2	PubMed	Pioglitazone and NAFLD	159
3	Embase	SGLT-2 Inhibitors and NAFLD	53
4	Embase	Pioglitazone and NAFLD	961
5	Wiley Online Library	SGLT-2 Inhibitors and NAFLD	206
6	Wiley Online Library	Pioglitazone and NAFLD	845

Inclusion and Exclusion Criteria

We have selected randomized controlled trials and cohort studies published within the last three years that meet specific criteria. These criteria include: (1) the study design being an RCT or cohort study, (2) the subjects having a clinical diagnosis of NAFLD/NASH, (3) the intervention drug being either an SGLT2 inhibitor or pioglitazone, and (4) the study reporting results for changes in liver steatosis, liver enzyme, and liver fibrosis of the SGLT2 group or pioglitazone group in comparison to the control group. Additionally, we only considered studies that were free and fully published between 2021-2023. 

During the screening process, we excluded studies that did not meet the aforementioned criteria. This included: (1) studies that were summaries, brief reports, or meeting summaries or involved animal and cell experiments, (2) incomplete studies such as pilots or preliminary reports, and (3) duplicate publications or studies with similar information. We also excluded articles focused on the pediatric population (less than 18 years), letters, expert opinions, unpublished or gray literature, and papers in languages other than English from our study.

Analysis of Study Quality/Bias

**Figure 1:**
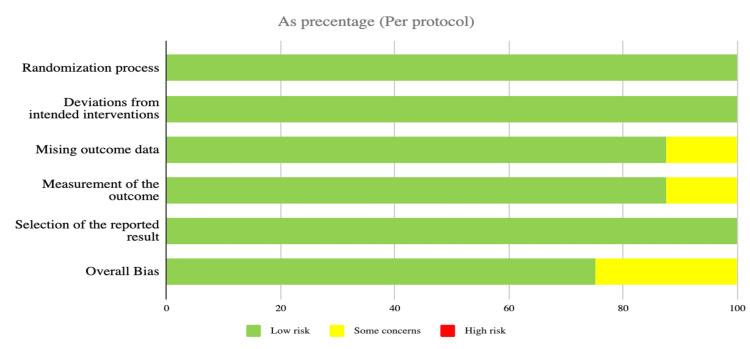
A quality check of RCT studies was done as per the Revised Cochrane Bias assessment tool for Randomized trials (Rob 2). All included RCTs were either good or fair in quality RCT: Randomized controlled trial; Rob 2: Risk-of-bias Version 2

We carefully assessed 18 studies for their quality using standardized evaluation tools. Out of them, nine studies met the criteria for medium or high quality and were included in the review (Figure 1). The assessment tools used were the Revised Cochrane Bias assessment tool for Randomized Controlled Trials (ROB 2) [10] and the Newcastle-Ottawa Scale for cohort studies [11]. The detailed overall scores and quality for each study are provided in Table 3 and Table 4.

**Table 3:** A quality check for cohort studies was done as per the Newcastle-Ottawa Scale a) A study can be awarded a maximum of one point for each category except for the comparability of cohorts based on design or analysis. b) Quality: >7/9 = HIGH, >5/9 = MEDIUM, <6/9 = LOW [11]

Categories	Euh W 2021 [12]
Representativeness of the exposed cohort	1
Selection of the non-exposed cohort	1
Ascertainment of exposure	1
Demonstration that outcome of interest was not present at the start of the study	0
Comparability of cohorts on the basis of the design or analysis	2
Assessment of outcome	1
Was follow-up long enough for outcomes to occur	1
Adequacy of follow-up of cohorts	1
Total	8
Quality	High

**Table 4:** A quality check of randomized controlled trial (RCT) studies was done as per the Revised Cochrane Bias assessment tool for Randomized trials (Rob 2) (x) High risk; (+) Low risk; (!) Some concerns (D1) Randomization process; (D2) Deviation from the intended interventions; (D3) Missing outcome data; (D4) Measurement of the outcome; (D5) Selection of the reported result

Author and Year	D1	D2	D3	D4	D5	Overall
Harrison SA, et al. 2022 [3]	+	+	+	+	+	+
Sayadishahrak M, et al. 2023 [1]	+	+	+	+	+	+
Takahashi H, et al. 2022 [6]	+	+	+	!	+	!
Yoneda M, et al. 2021 [8]	+	+	!	+	+	!
Cho KY, et al. 2021 [13]	+	+	+	+	+	+
Kamolvisit S, et al. 2021 [14]	+	+	+	+	+	+
Takeshita Y, et al. 2022 [15]	+	+	+	+	+	+
Chehrehgosha H, et al. 2021 [5]	+	+	+	+	+	+

Result

Our initial search of PubMed, Embase, and Wiley Online databases identified 2340 articles. Out of them, 1951 articles were removed after applying relevant filters as per our eligibility criteria (last three years, human studies, clinical trials, and randomized clinical trials), and 103 duplicates were excluded. The remaining 286 articles were screened by two individuals based on titles, abstracts, full text, and detailed inclusion-exclusion criteria. After the meticulous screening, the application of our inclusion criteria- randomized trials and cohort studies published in the English language in the last three years, and including free full-text papers relevant to our research question- we were left with 22 articles. Four articles could not be retrieved. A total of 18 studies were included for a thorough quality/bias assessment using standardized quality assessment tools. Nine studies were excluded after quality appraisal, and the final nine were included in this systematic review. We included one cohort that was high quality, as assessed by the Newcastle-Ottawa risk-of-bias tool [11]. We included eight randomized control trials in our analysis. Out of eight studies evaluated with the Revised Cochrane Risk of Bias tool for randomized trials (ROB 2) [10], six had a low risk, while two raised concerns.

**Figure 2:**
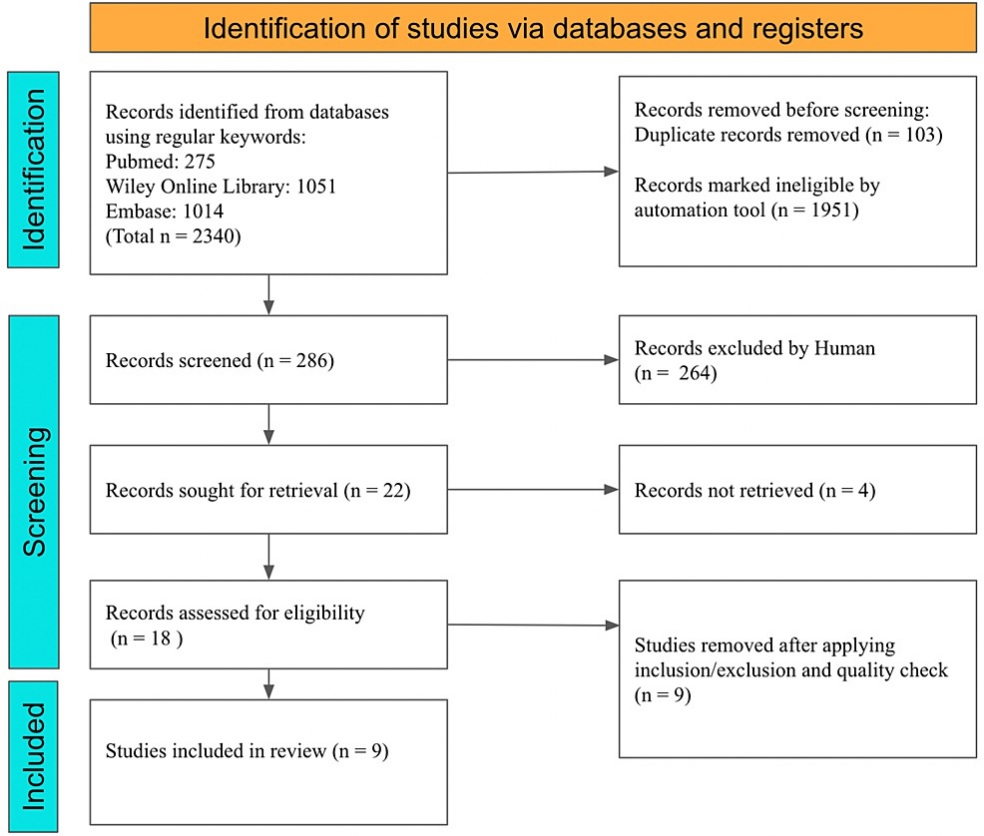
PRISMA flowchart of the literature and search strategy PRISMA: Preferred Reporting Items for Systematic Reviews and Meta-Analyses, n: number of studies

Figure 2 shows the PRISMA flowchart of the literature and the search strategy for the studies [9].

Characteristics of Included Studies

All the included studies were published between 2021 to 2023. From each of the selected articles, we have taken the following data: author name, study design, year of publication, type of intervention, follow-up period, sample size, inclusion and exclusion criteria, results, and conclusions. Table 5 shows the summary of the characteristics of the studies, which includes the inclusion and exclusion criteria and results.

**Table 5:** Summary of characteristics of included studies No.: Number; RCT: Randomized controlled trial; NAFLD: Non-alcoholic fatty liver disease; BMI: Body mass index; FBS: Fasting blood sugar; NASH: Non-alcoholic steatohepatitis; T2DM: Type-2 diabetes mellitus; CAP: Controlled attenuation parameter; HbA1c: Glycosylated hemoglobin; MRI: Magnetic resonance imaging; MRI-PDFF: Magnetic resonance imaging proton density fat fraction; GFR: Glomerular filtration rate; FLI: Fatty liver index; DAP: Dapagliflozin; PIO: Pioglitazone; HIV: Human immunodeficiency virus; SGLT2i: Sodium-glucose cotransporter 2 inhibitor; ALT: Alanine transaminase; g: Grams; mg: Milligrams; dB/m: decibel per meter; kg/m2: Kilogram per meters squared; mL/min: Milliliters per minute; m2: Meters squared

Author and year of publication	Type of Study	Intervention	Follow-up	No. cases/ control	Inclusion criteria	Exclusion criteria	Result and conclusion
Sayadishahraki M, et al. 2023 [1]	RCT	Pioglitazone	4 Months	22/22	Bariatric surgery candidates, Grade 3 NAFLD, BMI over 40 or 35 with diabetes or other comorbidities	Alcohol intake > 40 g for men, 20 g for women. Other liver diseases, liver cancer, taking hepatotoxic drugs.	Improvements in the stage of fatty liver, liver size, BMI, FBS, and lipid profile
Harrison. SA, et al. 2022 [3]	RCT	licogliflozin 30mg vs licogliflozin 150mg vs placebo	12 weeks	43/43/21	presence of NASH based on liver biopsy, phenotypic diagnosis of NASH	history or currently have liver diseases, cirrhosis, hepatic decompensation, severe liver impairment, type 1 diabetes, or uncontrolled diabetes	Patients with NASH who were treated with 150 mg of licogliflozin experienced a decrease in their serum alanine aminotransferase levels
Chehrehgosha H, et al. 2021 [5]	RCT	Empagliflozin vs Pioglitazone vs placebo	24 weeks	35/34/37	T2DM and NAFLD with controlled attenuation parameter (CAP) ≥ 238 dB/m in transient hepatic elastography	Type 1 diabetes, active or chronic hepatitis, cirrhosis and biliary disease, a history of consuming alcohol more than 20 g per day for women and 30 g per day for men, or taking medications associated with fatty liver	The empagliflozin group showed improvement in liver steatosis and fibrosis, while also experiencing a decrease in body weight and abdominal fat area
Takahashi H, et al. 2022 [6]	RCT	Ipragliflozin	72 weeks	21/25	Participants must be aged 20-80, diagnosed with NAFLD, have liver biopsy showing at least 5% steatosis in hepatic parenchyma	Those with diabetes complications, history of heart disease, heavy alcohol consumption, hepatitis B or C, and liver disease	Patients with NAFLD who undergo long-term treatment with ipragliflozin experience an improvement in their hepatic fibrosis
Yoneda M, et al. 2021 [8]	RCT	Tofogliflozin vs pioglitazone	24 weeks	21/19	People aged 20-74 with NAFLD, type 2 diabetes, HbA1c ≥6.5%, and high alanine transaminase levels	Viral hepatitis, alcohol intake men, more than 30 g/day; women, more than 20 g/day. Liver cirrhosis, taking vitamin E, contraindications to MRI	hepatic steatosis evaluated by MRI-PDFF, which showed a significant decrease in both groups
Cho KY, et al. 2021 [13]	RCT	Dapagliflozin (DAP) vs pioglitazone	24 weeks	27/26	Must be between 20-80 years old, have HbA1c levels between 6.5%-8.5%, a BMI of at least 23 kg/m2, and a GFR of 45 mL/min/1.73 m2 or above	habitual drinkers. Participants with an Fatty Liver Index (FLI ) <30	The DAP group experienced a greater decrease in FLI compared to the PIO group. This suggests that dapagliflozin could be a more advantageous treatment for patients with NAFLD than pioglitazone
Kamolvisit S, et al. 2021 [14]	RCT	pioglitazone vs placebo	48 weeks	49/49	HIV-positive individuals who were 35 to 65 years of age, prediabetes, NAFLD	hepatitis virus co-infection, critical illness, opportunistic infection; active alcohol intake (male > 30 g/day, female > 20 g/day), were pregnant or breastfeeding; contraindication for pioglitazone therapy	Pioglitazone significantly reduces liver stiffness, HIV population with prediabetes, and NAFLD
Takeshita Y, et al. 2022 [15]	RCT	Tofogliflozin	48 week	20/20	The liver biopsy specimens indicate a "definite" case of NAFLD in an individual with type 2 diabetes who is at least 20 years of age	liver problems, including viral infections, autoimmune hepatitis, primary biliary cirrhosis, and the use of drugs that can lead to fat accumulation in the liver or excessive alcohol intake	Tofogliflozin treatment improved liver health and metabolism by reversing specific markers related to energy metabolism, inflammation, and fibrosis in liver tissue
Euh W, et al. 2021 [12]	Cohort	SGLT2i vs placebo	9 months	100/150	age >18 years, presence of fatty liver by ultrasound	treatment with a glucagon like peptide-1 receptor agonist or insulin, chronic liver diseases other than NAFLD, alcohol consumption (daily intake >30 g for men and >20 g for women), or malignancies	SGLT2i decreased body weight and ALT level

Discussion

The purpose of this systematic review is to compare the effectiveness and safety of two types of medications - SGLT2 inhibitors and pioglitazone - in the treatment of NAFLD or NASH. NAFLD is characterized by the presence of at least 5% lipids in liver cells, without any evidence of damage caused by alcohol or drug abuse or any other condition that could lead to liver steatosis [16]. NASH, on the other hand, is defined as the presence of at least 5% lipids in liver cells along with signs of inflammation and damage to hepatocytes, with or without fibrosis. NASH can progress to liver cirrhosis, liver failure, or even the development of hepatocellular carcinoma [17]. Patients with NAFLD commonly have insulin resistance (IR) that enhances lipolysis from adipose tissue. Liver biopsy in NAFLD shows hepatic steatosis without inflammation or hepatocellular injury (hepatocyte ballooning); however, between 10% and 25% of patients with NAFLD show inflammatory infiltration leading to NASH. Approximately 25% of patients with simple steatosis may progress to NASH in three years [18]. Research has proposed that the development of NASH occurs in two stages. The first stage involves the buildup of fat in the liver, which can lead to increased insulin resistance. The second stage involves changes at the cellular and molecular level, including oxidative stress and oxidation of fatty acids in the liver. These changes can be caused by a variety of factors, such as cytokine injury, hyperinsulinemia, hepatic iron and/or lipid peroxidation, variation of the extracellular matrix, energy homeostasis, and alterations in the immune system function [19]. The use of pharmacological agents to treat NAFLD/NASH has significantly increased in recent years. This is due to the need for effective therapies to manage these conditions. The review aimed to assess whether SGLT2 inhibitors or pioglitazone provide superior outcomes in terms of efficacy and safety. Based on our analysis, we have discovered that SGLT2 inhibitors and pioglitazone are promising treatments for NAFLD/NASH. SGLT2 inhibitors have demonstrated an improvement in hepatic fibrosis through both pathological examination and surrogate markers. Studies included in this review have shown that SGLT2 inhibitors can reduce liver inflammation and prevent the progression of liver fibrosis. SGLT2 inhibitors reduce liver fat and improve metabolic aspects like glycemic control. Pioglitazone improves liver fat and metabolic parameters.

Mechanism of Action

SGLT2 inhibitors: SGLT2 inhibitors are a recent type of medication used to treat diabetes. They work by preventing the reabsorption of glucose in the kidneys, which results in more glucose being excreted in the urine [8]. When the kidneys are functioning normally and blood sugar levels are within the normal range, the renal filtration threshold for glucose is 180 g/day [17]. If plasma glucose levels exceed this threshold, it is eliminated from the body through the urine and sodium [17]. There are two types of cotransporters, SGLT1 and SGLT2. SGLT1 is found in both the intestine and the proximal convoluted tubule below the Bowman's capsule, while SGLT2 is exclusively present in the renal tubule. SGLT2 is responsible for reabsorbing around 97% of the glucose upstream of the proximal tubule, while the remaining 3% is reabsorbed downstream by SGLT1. In hyperglycemic states, the kidneys increase their renal resorption capacity to up to 600 g/day to prevent the loss of glucose through the urine. The SGLT cotransporter plays a key role in helping the body absorb glucose, sodium, and fluids. Blocking this transporter can help control blood sugar levels, balance sodium levels, and reduce water retention. SGLT2 inhibitors were specifically developed to manage T2DM by inhibiting glucose reabsorption in the kidney and decreasing blood glucose levels [17]. New studies have revealed that they can improve liver function for people with NAFLD and T2DM [5]. SGLT-2 inhibitors may help treat NAFLD/NASH by reducing insulin resistance and improving glucose control, thereby lowering hepatic fat accumulation and inflammation, although their exact mechanism in this context is still being studied. In addition, they have been suggested as a treatment for managing T2DM and stubborn ascites in cirrhosis due to their reliable effectiveness in promoting natriuresis and diuresis. For the reason mentioned, SGLT2 inhibitors show promise as treatment for patients with NAFLD.

Pioglitazone: Pioglitazone is a type of medication that falls under the thiazolidinedione class. Its main function is to improve insulin sensitivity in peripheral tissues. By decreasing insulin resistance in the body's tissues, this process assists in improving glucose uptake by peripheral tissues and reducing liver glucose output [1,20].

The mechanism of action of pioglitazone involves its interaction with nuclear receptors known as peroxisome proliferator-activated receptors gamma (PPARγ) found in adipose tissue, skeletal muscle, and liver cells. When pioglitazone binds to PPARγ receptors, it activates them, leading to several effects on glucose and lipid metabolism. Activating PPARy receptors by pioglitazone does the following: It improves insulin sensitivity, increases glucose uptake and its utilization, reduces hepatic glucose production, and modifies lipid metabolism. By increasing the uptake of fatty acids from the bloodstream into adipose tissue and skeletal muscle, pioglitazone affects lipid metabolism, which in turn helps to decrease circulating levels of free fatty acids and triglycerides. Pioglitazone also promotes the storage of fatty acids as triglycerides in adipose tissue, thereby reducing the release of fatty acids into the bloodstream.

The review systematically searched and analyzed relevant studies comparing the effects of SGLT2 inhibitors and pioglitazone on NAFLD/NASH. Efficacy outcomes primarily focused on liver fat content, liver enzyme levels, histological improvement, and metabolic parameters. Safety outcomes assessed adverse events, cardiovascular events, and other relevant complications.

Efficacy

A multicenter, open‐label, prospective recent randomized study conducted by Cho et al. in 2021 has suggested that dapagliflozin may be more beneficial than pioglitazone for treating patients with NAFLD [13]. This analysis involved 53 participants who had NAFLD, with 27 taking dapagliflozin and 26 taking pioglitazone. Fatty liver index (FLI) decreased significantly more in the dapagliflozin (DAP) group (58.3 ± 18.3 to 48.8 ± 19.5) compared with the PIO group (58.4 ± 20.6 to 61.2 ± 20.8) after 24 weeks (P < 0.01). The Fibrosis‐4 (FIB4) index was significantly decreased in the DAP group (1.37 ± 0.59 to 1.20 ± 0.50) compared with the PIO group (1.32 ± 0.50 to 1.35 ± 0.52; P < 0.01). Aspartate aminotransferase remained unchanged [13].

A study conducted by Takahashi et al. in 2022 found that the long-term use of ipragliflozin improved hepatic fibrosis in patients with NAFLD. Participants were given ipragliflozin (50 mg/day for 72 weeks), while those not taking SGLT2is, pioglitazone, glucagon-like peptide-1 analogs, or insulin (the control group) were compared based on changes in glycemic control, obesity, and liver pathology. At baseline, 81% and 72% of participants in the ipragliflozin and control groups respectively had hepatic fibrosis. This improved in 70.6% of participants in the ipragliflozin group and 22.2% in the control group, which was statistically significant. In addition, 66.7% of the ipragliflozin-treated group had their NASH resolved compared to 27.3% in the control group. None of the participants in the ipragliflozin group developed NASH, while 33.3% in the control group did. The activity levels of aspartate aminotransferase, alanine aminotransferase, and gamma-glutamyl transferase were significantly lower in the ipragliflozin group than baseline, whereas there were no significant changes in the control group. The differences in gamma-glutamyl transferase activity changes between the two groups showed significant changes at 24, 48, and 72 weeks into the study. The ipragliflozin group also had a higher proportion of patients with at least a one-score or one-stage reduction in ballooning (52.4%) and fibrosis (57.1%) after 72 weeks of treatment compared to the control group (24% for ballooning and 16% for fibrosis) [6].

In 2021, Chehrehgosha et al. conducted a study involving 106 patients, which indicated that treating NAFLD and T2DM patients with empagliflozin for 24 weeks resulted in a better improvement of liver steatosis and fibrosis compared to pioglitazone. A significant reduction in the placebo-corrected change in liver stiffness measurement (LSM) was found with empagliflozin compared to pioglitazone: - 0.77 kPa (- 1.45, - 0.09), p = 0.02, versus 0.01 kPa (95% CI - 0.70, 0.71, p = 0.98), p for comparison = 0.03. No significant differences were found between the two treatment groups in terms of changes in the serum levels of aspartate aminotransferase and alanine aminotransferase. Additionally, there were no significant changes observed in the FIB4 index, NAFLD fibrosis score, and aspartate aminotransferase to platelet ratio index (APRI) [5].

Licogliflozin can selectively and strongly block SGLT1 and SGLT2. Therefore, a phase 2a study was conducted in 2022. The study used randomized and double-blind methods. The study with 96 patients included placebo (n = 21), licogliflozin 30 mg (n = 43), and licogliflozin 150 mg (n = 43). Biomarkers of liver fibrosis were assessed. Licogliflozin treatment resulted in a statistically significant reduction in enhanced liver fibrosis (ELF) score (geometric mean ratio to baseline) of 4% (P = 0.038) with licogliflozin 30 mg and 5% (P = 0.022) with licogliflozin 150 mg when compared with placebo. Similar decreases were noted in the amino-terminal propeptide of procollagen type III (PIIINP) with 30 mg (19%; P = 0.025) and 150 mg (19%; P = 0.038) compared with placebo. Likewise, the tissue inhibitor of matrix metalloproteinase-1 (TIMP1) decreased for the 30 mg and 150 mg doses was 5% (P = 0.151) and 12% (P = 0.001), respectively, compared with placebo [3].

In 2020-2021, a study was conducted on 44 patients who had grade 3 NAFLD. The patients were given pioglitazone 15 mg tablets twice a day for four months in a randomized controlled trial. After the treatment, it was observed that 50% of the patients in the pioglitazone group had improved to grade 1 NAFLD, while 50% of the patients in the other group showed improvement to grade 2 NAFLD. The results showed significant improvements in the patients (P < 0.001). In addition, the intervention group showed significant improvements in various factors such as liver size, size of the left liver lobe, fasting blood sugar (FBS), alanine aminotransferase (ALT), and BMI. However, no notable advancements were observed in the control group [1]. Pioglitazone has been found to significantly decrease transient elastography (TE), controlled attenuation parameter (CAP), and liver stiffness in individuals with prediabetes and NAFLD who are also HIV-positive [14].

A clinical trial lasting 48 weeks was conducted on participants with biopsy-confirmed NAFLD. The trial used randomization and parallel grouping and yielded interesting results. The tofogliflozin group showed significant improvement in fibrosis scores (60%, P = 0.001) and the histological variables of steatosis (65%, P = 0.001). Moreover, the trial also showed improvement in hepatocellular ballooning (55%, P = 0.002), and lobular inflammation (50%, P = 0.003) [15]. Another study, a nine-month cohort study conducted in 2021 comparing SGLT2i vs placebo involving 100 and 150 participants, respectively, concluded that SGLT2i decreased ALT levels and body weight [12].

A study was conducted on 40 patients with T2DM and NAFLD to compare the effectiveness of tofogliflozin and pioglitazone treatments. The patients were given either 20 mg of tofogliflozin or 15 mg - 30 mg of pioglitazone orally once every day for 24 weeks. MRI was used to evaluate changes in hepatic steatosis after 24 weeks of treatment, and it was found that there was a significant decrease in both groups. The pioglitazone group showed a decrease of -7.54% and the tofogliflozin group showed a decrease of -4.12% in proton density of the fat fraction (MRI-PDFF). The results showed no statistically significant difference between the low-dose pioglitazone and tofogliflozin groups. However, the pioglitazone group tended to be more effective in hepatic steatosis. The study also evaluated the effects of tofogliflozin and pioglitazone on liver stiffness using MRE. It was found that during the 24-week treatment, pioglitazone greatly enhanced the measurement of liver stiffness using MR elastography (MRE-LSM). On the other hand, tofogliflozin did not result in a decrease in MRE-LSM. However, it is important to note that due to the short duration of the study, changes in fibrosis, steatosis, and inflammation may have impacted the variation in MRE values. Therefore, long-term testing is recommended when evaluating improvements in liver fibrosis [8].

After reviewing the data, we have found that SGLT2 inhibitors and pioglitazone are promising treatments for NAFLD/NASH. SGLT2 inhibitors have shown improvement in hepatic fibrosis through both pathological examination and surrogate markers. Although the mechanism behind this improvement is unclear, there is evidence that SGLT2 inhibitors can reduce liver inflammation and prevent the progression of liver fibrosis in mouse models of non-alcoholic steatohepatitis and diabetes [13]. The use of SGLT2 inhibitors has been linked to lower liver fat content and enzyme levels. These inhibitors have also been found to enhance metabolic factors, including glycemic control, weight management, and blood pressure. In addition, pioglitazone has also been shown to be effective in improving liver fat content, histological features, and metabolic parameters. 

Regarding safety, SGLT2 inhibitors were generally well-tolerated. There was a low risk of hypoglycemia, and weight loss was observed as a beneficial side effect. However, concerns regarding the risk of diabetic ketoacidosis and genital infections should be taken into account [5]. Pioglitazone, while effective, may be associated with adverse events such as weight gain, fluid retention, and a potentially increased risk of heart failure, which should be monitored closely [8].

Limitation

Limited numbers of high-quality studies are available. This restricts the scope and comprehensiveness of the systematic review. The available studies have a relatively short duration, making it difficult to assess the long-term efficacy and safety of SGLT2 inhibitors and pioglitazone in NAFLD or NASH treatment. Restricting the search to English articles can lead to overlooking relevant studies published in other languages.

## Conclusions

In conclusion, SGLT2 inhibitors and pioglitazone can be potential treatment options for NAFLD/NASH. Choosing between SGLT2 inhibitors and pioglitazone should not be a one-size-fits-all approach. The decision must consider various factors, such as the patient's underlying medical conditions, preferences, and safety profile. 

While both SGLT2 inhibitors and pioglitazone show promise, more extensive and long-term research is essential to establish their efficacy and safety for NAFLD/NASH conclusively. We considered studies that were free, only in the English language, and fully published between 2021- 2023. Future studies should focus on larger patient populations and longer follow-up periods to assess their impact on liver-related outcomes, including fibrosis regression and the prevention of disease progression. Additionally, more comprehensive data will help healthcare providers make informed decisions about the most appropriate treatment for individual patients.
